# University of Buffalo

**Published:** 1883-08

**Authors:** 


### UNIVERSITY OF BUFFALO.

Dr. E. M. Moore, of Rochester, N. Y., who has for so many years been the Professor of Surgery in the Medical Department of the University of Buffalo, has resigned his position, and will be succeeded by Dr. Roswell Park, of Chicago, the editor of *The Weekly Medical Review.*

Dr. Moore possesses a national reputation as a surgeon, and has been remarkably successful in many capital operations. At its late meeting he received the honor of election to the Presidency of the American Surgical Association. As a lecturer he has few equals, and no superiors. Gifted with an unusual command of language, he is at once concise and entertaining. He is thoroughly systematic in his lectures, and his points are so clearly made and so aptly illustrated that they are certain to remain permanently fixed in the memories of even careless students. He has, withal, such a fund of apposite anecdotes, and is so thoroughly cheerful and buoyant in his manner, so earnest and enthusiastic in his teaching, that no matter how much the lectures of other professors were shirked, Prof. Moore was certain to meet the faces of the whole class. In common with thousands of others of the Alumni of the institution with which he has for so many years been connected, we sincerely regret that the advance of years and the necessities of a great practice have forced him to resign the position which he has so highly honored.

If anything could console the friends of the Buffalo University, it is the fact that Prof. Moore is to be succeeded by one who bears such a high reputation as does Prof. Park, and the Faculty are to be congratulated upon his accession. That his career may be as long and as prosperous as that of his predecessor, is the earnest wish of every graduate.

